# Healing effects of monomer and dimer resveratrol in a mouse periodontitis model

**DOI:** 10.1186/s12903-022-02499-2

**Published:** 2022-11-01

**Authors:** Eri Ikeda, Daiki Tanaka, Michael Glogauer, Howard C Tenenbaum, Yuichi Ikeda

**Affiliations:** 1 Department of Periodontology, Graduate School of Medical and Dental Sciences, Tokyo Medical and Dental University, 1-5-45 Yushima, 113-8549 Bunkyo, Tokyo, Japan; 2 Faculty of Dentistry, University of Toronto, 124 Edward St, M5G 1G6 Toronto, ON Canada; 3 Department of Dental Oncology and Maxillofacial Prosthetics University Health Network - Princess Margaret Cancer Centre, 610 University Avenue, M5G 2M9 Toronto, ON Canada; 4 Centre for Advanced Dental Research and Care, Department of Dentistry, Mount Sinai Hospital, 600 University Avenue, M5G 1X5 Toronto, ON Canada; 5 Department of Molecular Immunology, Graduate School of Medical and Dental Sciences, Tokyo Medical and Dental University, 1-5-45 Yushima, 113-8549 Bunkyo, Tokyo, Japan

**Keywords:** Resveratrol, Melinjo seed extract, Gnetin C, Anti-inflammatory agents, Inflammation, Periodontitis, Reactive oxygen species

## Abstract

**Background:**

The antioxidant and anti-inflammatory effects of resveratrol have been reported previously. Particularly, monomeric trans-resveratrol has been demonstrated to produce positive effects in various pathological processes. We reported previously that resveratrol dimer-rich melinjo extract, among others, caused bone healing, decreased local oxidative damage, and activated antioxidants nuclear factor erythroid 2-related factor 2 (Nrf2) pathways in a mouse model of experimentally induced periodontitis (EP). This study aimed to compare the bone-healing effects of the resveratrol monomer to the resveratrol dimer (gnetin C found in melinjo seed extract) in a model of EP and investigate the involvement of Nrf2 for effects of either form of resveratrol.

**Methods:**

EP was induced experimentally in mice by placement of a 9 − 0 silk ligature around the left second molar. Mice received 10 mg/kg of either resveratrol monomer or dimer intraperitoneally on day 15 after induction of EP. The bone level around the ligated teeth was measured over time, and levels of proinflammatory cytokines and oxidative stress were measured in the periodontal tissues around the ligated teeth.

**Results:**

Resveratrol dimer induced greater periodontal bone healing as compared to that related to use of the resveratrol monomer. It appears that healing of periodontal bone in either group was likely related to master regulation of antioxidant nuclear factor erythroid 2-related factor 2 (Nrf2) significantly. Downregulation of IL-1β, a proinflammatory cytokine was also demonstrated in the resveratrol dimer group.

**Conclusion:**

Our results showed that administration of resveratrol in either dimer form or the monomeric form reduced periodontal bone loss with greater inhibition of bone loss being demonstrated in the dimer group as compared to the monomer group and that these effects were related in all likelihood to decreased oxidative stress and hence reduction in local inflammation.

## Backround

Periodontitis is a common oral inflammatory disorder in which imbalance of oral microbiota initiates the host immune response leading periodontal disruption [1, 2]. It has complex etiological factors including environmental, occlusal, and genetic factors [3, 4]. Periodontitis may not only cause tooth-supporting tissue destruction, but is also associated with systemic diseases including diabetes [5] and cancer [6]. Resveratrol (3, 4′, 5-trihydroxy-trans-stilbene), a natural polyphenol present in different plant species, is considered to have a wide array of possible health benefits. In 2006, the discovery of its lifespan extending effects [7] brought much attention to this molecule. Resveratrol has also been associated with anti-oxidative [8], anti-cancer [9, 10], cardio-protective [11], anti-inflammatory [12], anti-diabetic [13], anti-microbial [14] effects. There are several sources of resveratrol, including melinjo (*Gnetum gnemon L.*) seed extract (MSE). Melinjo is a native plant to Indonesia; MSE, a readily available dietary supplement, is found commonly in Southeast Asian diets [15]. MSE contains trans-resveratrol (resveratrol monomer) and several resveratrol containing compounds, including gnetin C (resveratrol dimer) and gnetin C’s derivatives (gnemonoside A and D [resveratrol dimer glycosides]) [15, 16]. Approximately 99% of the resveratrol found in MSE is in the dimer form (gnetin C) dimer and its derivatives. Whereas resveratrol dimer is maintained in plasma over 96 h, its monomer is metabolized within 24 h [17]. Although monomeric trans-resveratrol has been demonstrated to have positive biological effects in various pathological processes, it is still unknown whether its dimeric or polymeric forms have similar or even more potent effects.

Resveratrol inhibits the progression of periodontal tissue destruction and decreases oxidative stress in animal models of experimental periodontitis [18–20]. Moreover, we have previously shown in a mouse model of experimental periodontitis (EP) that treatment with MSE accelerates bone healing, and can even reverse bone loss (even when the etiologic factors used in EP remain present) and that these effects are related to decreases in local oxidative stress-related damage and upregulated the master regulation of antioxidants nuclear factor erythroid 2-related factor 2 (Nrf2) activity [21]. Our previous study evaluated the effects of a mixture of several resveratrol forms (monomer, dimer, and dimer derivatives) in EP. Since then, new technological advances have allowed for the purification of gnetin C, the resveratrol dimer, from MSE.

Therefore, we aimed to compare the ameliorative potential of resveratrol dimer (gnetin C) and resveratrol monomer on periodontal bone loss in EP. We hypothesized that treatment with gnetin C would be associated with reduced periodontal tissue destruction and that these effects would be more potent than those observed using resveratrol in its monomeric form. Moreover, we anticipated that treatment of EP with either form of resveratrol should be related to downregulation of proinflammatory cytokines, oxidative stress, and mediated through the Nrf2 pathway.

## Methods

### Animals and experimental design

C57BL/6 N wild-type mice were obtained from CLEA Japan, Inc. (Tokyo, Japan). The Nrf2^−/−^ mice bred on a C57BL/6J background, were purchased from RIKEN BioResource Center (Ibaraki, Japan). All mice were aged 6 weeks. EP was induced as described previously by placing a silk ligature around the left second molar until end of the experiment. The right molar was used as a control site (no EP) as described previously [21, 22]. After adequate induction of EP, we injected intraperitoneally either gnetin C or resveratrol monomer (10 mg/kg body weight) on day 15. Mice were euthanized after 7 to 8 days after injection of either form of resveratrol. The persistence of the ligatures was checked at the day of euthanization and mice without ligatures (i.e., the ligatures had been lost) were excluded from the study.

### Microcomputed tomography analysis

Microcomputed tomography (µCT) was performed with an X-ray CT (R_mCT2, Rigaku Corporation, Tokyo, Japan). Wild-type mice were scanned at 0, 2, 6, and 8 days after injection of the different resveratrol forms. The Nrf2^−/−^ mice were scanned at 0-, 4-, and 7-days following injection of gnetin C or resveratrol monomer. After image standardization, bone loss was evaluated using imaging software, (Image J; National Institutes of Health, Bethesda, MD) [23] on selected slices in orthogonal views of the image stack acquired from the mesial and distal cementoenamel junction to the alveolar bone crest.

### Quantitative reverse transcription-polymerase chain reaction

Total RNA was extracted from wild-type mouse gingival tissue 8 days after resveratrol injection and complementary DNA was generated as described previously [21]. The gingival tissue of the palatal aspects of the ligated left second molar was used whereas the right second molar served as a healthy control. Quantitative reverse transcription-polymerase chain reaction (qRT-PCR) was performed in duplicate. Glyceraldehyde 3-phosphate dehydrogenase (GAPDH) was used to normalize the expression of the tested genes (IL-1β, IL-6, and TNF-*α*).

### Immunohistochemistry

Immunohistochemical staining of tissue sections obtained from wild-type mice at the Biopathology Institute Co., Ltd. was performed as per routine (Oita, Japan). A novel biomarker for oxidative stress, 8-hydroxy-2′-deoxyguanosine (8-OHdG), was identified to evaluate reactive oxygen species (ROS) expression at the ligated sites. A primary mouse anti-8-OHdG (JaICA, clone N45.1, Shizuoka, Japan), a universal LSBA2 visualization kit (Agilent, K0675, Santa Clara, CA, United States), and hematoxylin counterstaining were used for probing and staining.

### Statistical analysis

All statistical analyses were performed with EZR statistical software (Saitama Medical Center, Jichi Medical University, Saitama, Japan). Welch’s t-test or Tukey’s HSD test were performed as specified. The Shapiro–Wilk test was used to test for normality. Bartlett’s test was used to test for homogeneity of variances. The threshold for statistical significance was set at p < 0.05. Error Bars were displayed that represented the standard deviation of a data set. The sample size was estimated based on the results of a previous study [16, 17]. A minimum of three samples per group were required to detect a significant difference with an alpha level of 0.05 and a power of 0.80.

## Results

### Greater healing of bone was seen in gnetin C group at sites with periodontitis

**Fig. 1:**
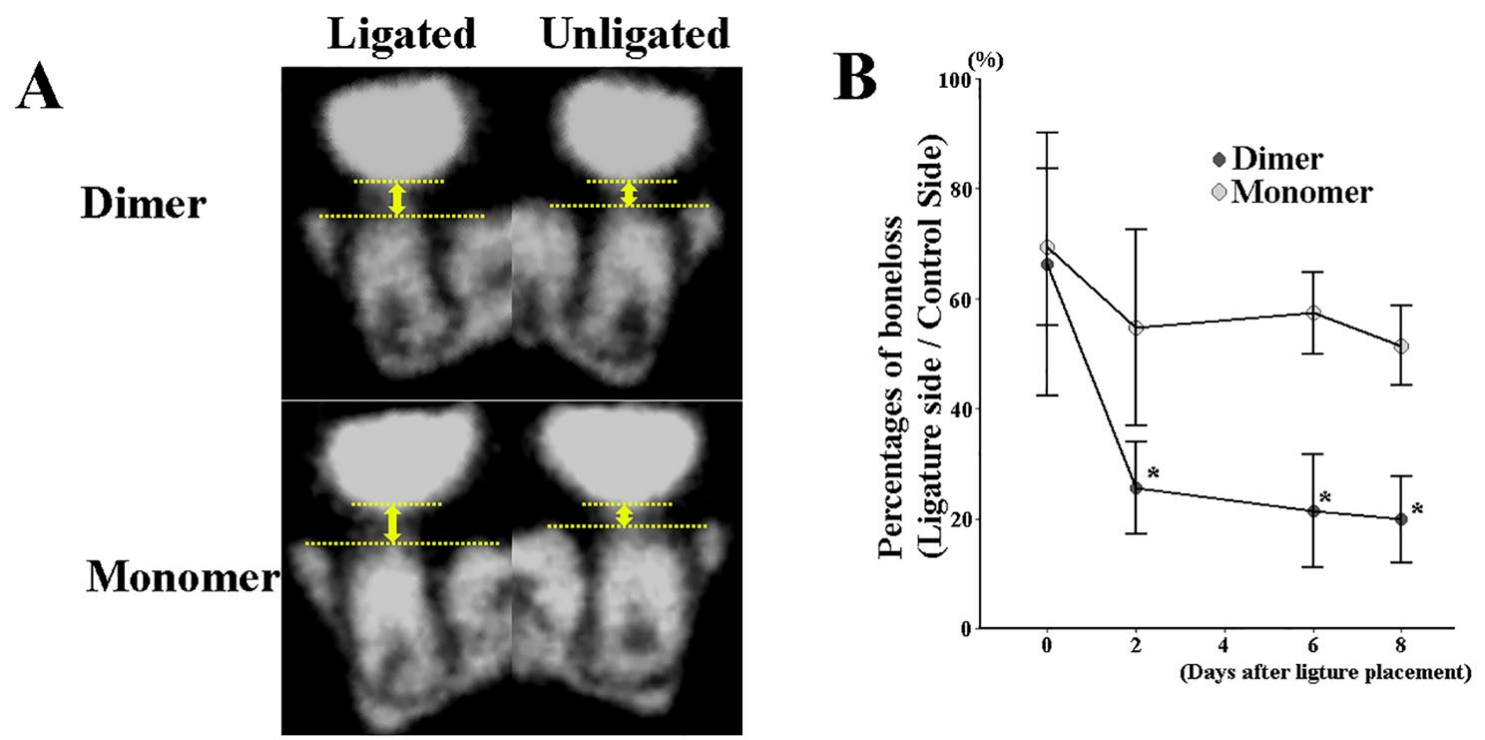
**Micro-CT image analysis of alveolar bone in wild-type mice**. **(A)** Bone loss was measured by the distance from the cement–enamel junction to the alveolar bone crest (yellow arrow). **(B)** The length of bone loss of mesial and distal sites was measured 2, 6, 8 days after resveratrol (either gnetin C or monomer) injection. Data are presented as mean ± standard deviation. *P < 0.05, **P < 0.01

The bone level at sites with established periodontitis in wild-type mice is shown in Fig. 1. Micro-CT imaging showed no significant differences in bone loss between groups on the day of resveratrol administration (day 15) suggesting equal disease loads prior to resveratrol treatment in all groups. Bone healing was observed over time with both resveratrol monomer and gnetin C and this occurred, as noted above, with ligatures still in place. Significantly greater bone regeneration was demonstrated at all evaluated timepoints (2, 6, 8 days after resveratrol injection) in the gnetin C group as compared to that which occurred in the resveratrol monomer-treated groups.

### Treatment with resveratrol dimer inhibits production of IL-1β

**Fig. 2:**
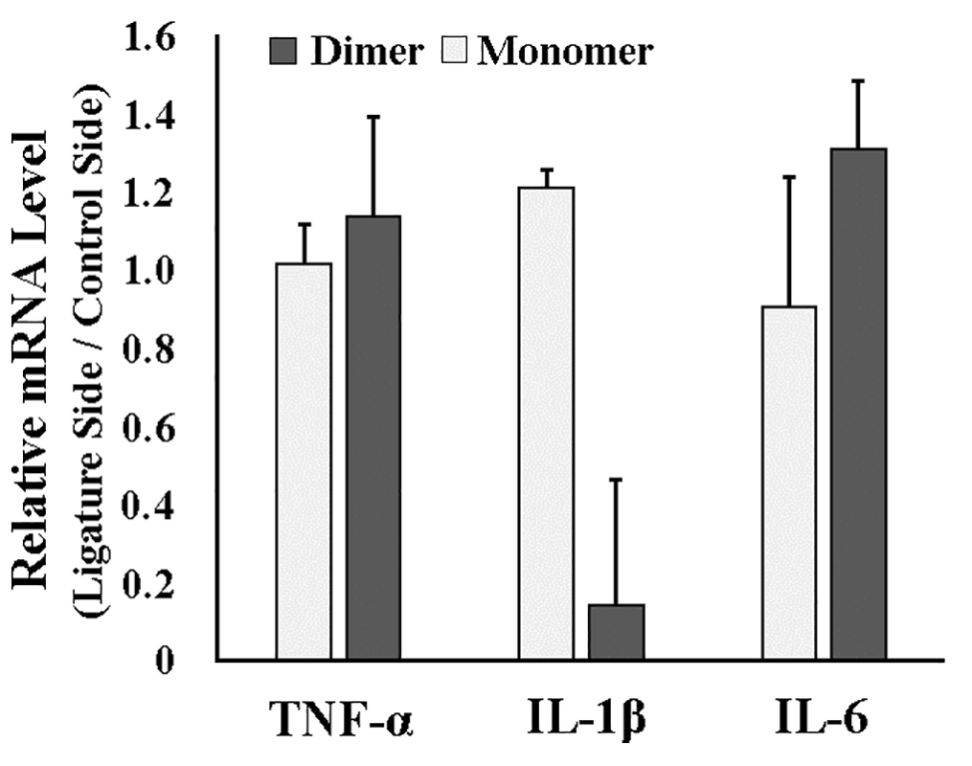
**Effects of resveratrol (either gnetin C or monomer) administration on proinflammatory cytokine gene expression in gingival tissue of wild-type mice**. mRNA levels of proinflammatory cytokines (IL-1β, IL-6, and TNF-α) in gingival tissue of wild-type mice determined using qRT-PCR. Data are presented as mean ± standard deviation. *P < 0.05

We evaluated the expression levels of known proinflammatory cytokines associated with periodontitis (IL-1β, IL-6, and TNF-α). IL-1β mRNA expression levels were significantly lower in the gnetin C group than in the resveratrol monomer group (*P* < 0.05). However, there were no significant differences in IL-6 or TNF-α levels between groups (Fig. 2).

### Both resveratrol monomer and dimer downregulate oxidative stress at the ligated sites

**Fig. 3:**
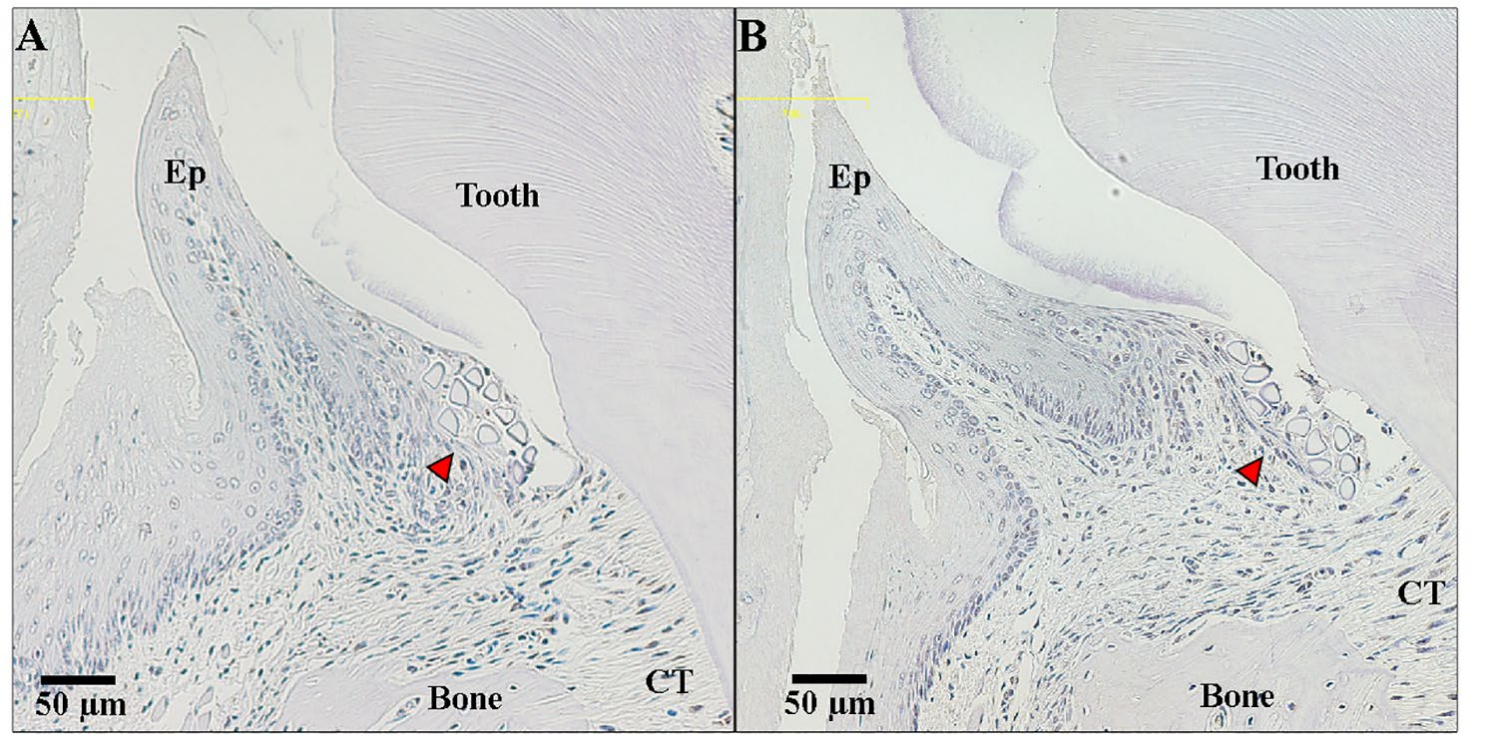
**Histological assessment of wild-type mice maxillae 8 days after resveratrol (either gnetin C or monomer) administration**. Reactive oxygen species damage in periodontitis lesions surrounding the ligature (red arrowheads) was assessed by immunohistochemistry for 8-hydroxydeoxyguanosine (8-OHdG), a specific marker for DNA oxidative damage

To observe the antioxidant effects of resveratrol, we visualized ROS expression at sites with periodontitis. There were few 8-OHdG-positive cells around the ligated molars of gnetin C and resveratrol monomer mice (Fig. 3).

### Treatment with resveratrol monomer and dimer did not induce bone healing in Nrf2^−/−^ mice

**Fig. 4:**
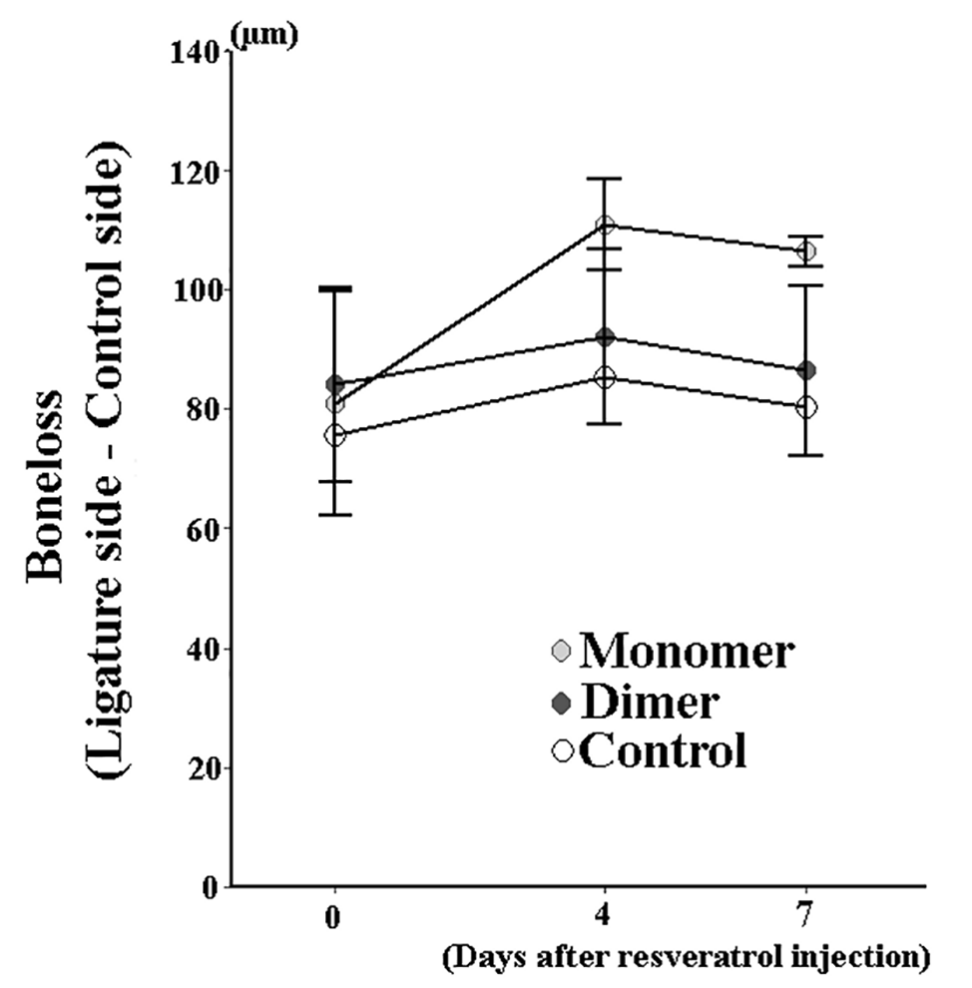
**Micro-CT image analysis of alveolar bone in Nrf2**^**−/−**^**mice**. Bone loss was measured by the distance from the cement–enamel junction to the alveolar bone crest. The length of bone loss of mesial and distal sites was measured 4 and 7 days after resveratrol injection (either gnetin C or monomer resveratrol). Data are presented as mean ± standard deviation. No significant difference was observed among resveratrol monomer, gnetin C (dimer), and control groups

To acquire additional insight into the effects of resveratrol on periodontal tissue breakdown, alveolar bone loss was measured by µCT in Nrf2^**−/−**^ mice. All groups presented observable bone loss at sites with EP. Statically significant alveolar bone loss was not measured in the resveratrol monomer, gnetin C, and control groups (Fig. 4). There were no observable differences in bone loss amongst these groups.

## Discussion

The current study demonstrates the effect of resveratrol dimer and monomer on the healing of EP in mice and that the healing-inducing effects were likely related to oxidative stress and mediated via that antioxidant Nrf2 pathway. Moreover, resveratrol dimer induced greater periodontal bone healing as compared to that related to use of the resveratrol monomer. We have shown previously [21] that treatment with MSE (a mixture of resveratrol monomer, dimer, and related complexes) promoted healing of periodontal bone loss in mice, even when the agents used to induce periodontitis were not removed (in this case the silk ligatures, which helped retain pathogenic biofilms). As expected, suture ligated mice not treated with either formulation of resveratrol continued to lose bone until the last experimental day. Similarly, our current study demonstrated the benefits of using a gnetin C instead of its monomeric form with regard to reduction of bone loss and in fact actual regeneration of lost bone, especially with the dimeric form of resveratrol. Treatment with gnetin C also decreased levels of a proinflammatory cytokine, IL-1β, in mice with EP. These findings also showed that positive effects on bone levels were more robust in animals treated with the gnetin C in comparison to the monomer. This said, the resveratrol monomer also induced bone healing which was demonstrated despite the ongoing presence of the disease-inducing silk-ligatures which retained the pathogenic biofilms but at a level that was less robust than that produced by the dimer. Previous pharmacokinetic studies have shown that gnetin C is maintained in plasma for > 96 h while trans-resveratrol is metabolized within 48 h [17] and these findings might explain the stronger effect of gnetin C as compared to monomeric resveratrol.

Current treatments of periodontitis aim at reducing microbial load through mechanical means. Although generally successful, these treatments involve significant and invasive procedures and the provision of interventions (e.g., gnetin C) might be highly assistive. This is borne out by our reported findings showing that resveratrol downregulates oxidative stress and level of IL-1β without any attempt to reduce microbial irritants that promote the progression of EP. It has been suggested that oxidative stress plays a critical role in periodontal bone loss and tissue damage [24, 25]. In addition to direct tissue destruction, ROS also modulate the immune-inflammatory system leading to tissue damage secondary to the induction of a proinflammatory state [26]. Additionally, we know that ROS-related oxidative stress and inflammation are also necessary for the development of the principal cells responsible for degradation of bone, osteoclasts, in presence of RANKL [27]. Thus, ROS neutralization by resveratrol should prevent osteoclast formation thereby preventing periodontal bone loss. Processing of proinflammatory cytokine IL-1β is regulated by nucleotide-binding oligomerization domain-like receptor protein 3 (NLRP3) inflammasome complex, and NLRP3 expression is upregulated in the gingival tissue of patients with periodontitis [28, 29]. Resveratrol is reported to inhibit NLRP3 inflammasome activation by suppressing mitochondrial damage [30]. Perhaps the NLRP3 inflammasome plays some role in the bone-healing effects of resveratrol.

Our current study demonstrated that bone healing did not occur in Nrf2 null mice with resveratrol treatment suggesting a crucial role of Nrf2 in relation to mechanisms related to the effects of resveratrol administration for EP. Nrf2 regulates intracellular redox balance and the antioxidants in the cell and thereby regulates inflammation. We reported previously that Nrf2 deficiency results in greater periodontal breakdown in mice with EP [31]. Notably, the Nrf2 antioxidant pathway is downregulated in oral peripheral blood polymorphonuclear neutrophils of patients with periodontitis [31]. This also explains why, in Nrf2 null mice, neither form of resveratrol could be expected to have any effects. Based on the above, resveratrol could reduce oxidative stress first by acting as an antioxidant through the Nrf2 antioxidant pathway and by quenching ROS activity directly while putatively inhibiting ROS production by inflammatory cells.

## Conclusion

The current study demonstrates the effects of resveratrol dimer and monomer on the healing of EP in mice and our results revealed the significance of the Nrf2 antioxidant pathway with respect to the periodontal bone-healing effects of resveratrol. Most notably, systemic administration of gnetin C caused higher periodontal bone healing in mice as compared to healing induced by the resveratrol monomer. Based on our results, reduced proinflammatory cytokine IL-1β was probably involved in the differential healing effects. These findings suggest that resveratrol, especially gnetin C, could be considered as a useful therapeutic addition, for the management of periodontitis.

## Data Availability

The datasets generated and/or analyzed during the current study are not publicly available but are available from the corresponding author on reasonable request.

## Abbreviations

EP: Experimentally-induced periodontitis
GAPDH: Glyceraldehyde 3-phosphate dehydrogenase
MSE: Melinjo seed extract
µCT: Microcomputed tomography
Nrf2: Nuclear factor erythroid 2-related factor 2
NLRP3: Nucleotide-binding oligomerization domain-like receptor protein 3
qRT-PCR: Quantitative reverse transcription-polymerase chain reaction
ROS: Reactive oxygen species
8-OHdG: 8-hydroxy-2′-deoxyguanosine
